# Acute Esophageal Necrosis (Gurvits Syndrome): A Rare Complication of Diabetic Ketoacidosis in a Critically Ill Patient

**DOI:** 10.1155/2020/5795847

**Published:** 2020-02-27

**Authors:** Linda P. Vien, Ho-Man Yeung

**Affiliations:** Department of Medicine, Temple University Hospital, 3401 North Broad Street, Philadelphia, PA 19140, USA

## Abstract

Acute esophageal necrosis (AEN) is a rare clinical diagnosis that primarily affects the distal third of the esophagus. AEN causes odynophagia, leading to decreased oral intake and food avoidance. AEN can arise in critically ill patients with multiple comorbidities and is an uncommon complication of diabetic ketoacidosis (DKA). We present a case of a young female with poorly controlled, insulin-dependent diabetes mellitus type 2 who developed odynophagia, small volume coffee-ground emesis, and inability to tolerate oral intake after resolution of DKA. She was found to have esophagitis with esophageal necrosis in the middle third of the esophagus on upper gastrointestinal endoscopy. She was subsequently treated with fluid resuscitation and gastric acid suppression and improved clinically with slow advancements in her diet. The location of her lesion in the more vascularized middle one-third of the esophagus and lack of significant blood pressure variations during her hospital stay make her case unique. Thus, AEN should be considered in the differential diagnosis for critically ill patients who present with vague symptoms such as odynophagia and gastrointestinal bleeding.

## 1. Introduction

Acute esophageal necrosis (AEN), also called black esophagus or Gurvits syndrome, is a rare clinical diagnosis that primarily affects the lower one-third of the esophagus. AEN is observed in 0.28% of all upper gastrointestinal endoscopies [1]. Patients often describe symptoms of chest pain and odynophagia, leading to poor oral intake and resultant food avoidance. The distal one-third of the esophagus is the most vulnerable to ischemia and necrosis because the proximal two-thirds have a denser vascular supply [2]. AEN is a rare and potentially life-threatening complication of diabetic ketoacidosis (DKA) and can arise in critically ill patients with multiple comorbidities.

## 2. Case Presentation

A 37-year-old Hispanic female with poorly controlled, insulin-dependent diabetes mellitus type 2, hyperlipidemia, hypertension, and gastroesophageal reflux disease (GERD) presented to the emergency department with lethargy, nausea, vomiting, decreased oral intake, and cold-like symptoms. She had been seen in the emergency room three days prior and was found to have a white blood cell count of 18.1 thousand/mm^3^, blood glucose of 118 mg/dL, HCO_3_^–^ of 14 mmol/L, anion gap of 13, and ketonuria. She was discharged home but returned to the emergency room due to persistent decreased oral intake and inability to take her home medications.

On presentation, she was afebrile with a blood pressure of 154/108 mmHg, respiratory rate of 21 breaths per minute, and heart rate of 120 beats per minute. On examination, she was somnolent and appeared uncomfortable. She was well-nourished but had dry mucous membranes. Her abdominal exam was unremarkable. Her laboratory findings revealed Na^+^ of 127 mg/dL, HCO_3_^–^ of 7 mmol/dL, anion gap of 23, creatinine of 1.29 mg/dL, and glucose of 426 mg/dL. Her complete blood count was remarkable for a leukocytosis of 29.1 thousand/mm^3^. Urinalysis was significant for ≥1000 mg/dL glucose and ≥80 mg/dL ketones without evidence of leukocyte esterase or nitrites. Arterial blood gas showed a pH of 7.20, pCO_2_ of 8 mmHg, pO2 of 160 mmHg, HCO_3_^–^ of 3 mEq/dL, and arterial oxygen saturation of 99%. Lactic acid was 2.4 mg/dL, and *β*-hydroxybutyrate was 57.6 mg/dL. Hemoglobin A_1_C was elevated to 10.8%. Electrocardiogram showed sinus tachycardia with heart rate of 117 beats per minute and prolonged QTc of 507. She was given two liters of intravenous normal saline boluses. She was admitted to the medical intensive care unit (ICU) and started on an insulin drip for DKA.

Within two days, she improved clinically with normalization of her metabolic derangements and was transitioned to her home dose of subcutaneous insulin. She tolerated a liquid diet and was transferred out of the ICU. The following day, she complained of severe chest pain associated with swallowing that was different from her usual GERD symptoms. She also had small volume coffee-ground emesis. She was unable to eat any food, solid or liquid, due to severe odynophagia. Cardiac troponin I and electrocardiogram were negative for cardiac ischemia. She had no improvement after two days of acid suppressive therapy.

**Figure 1:**
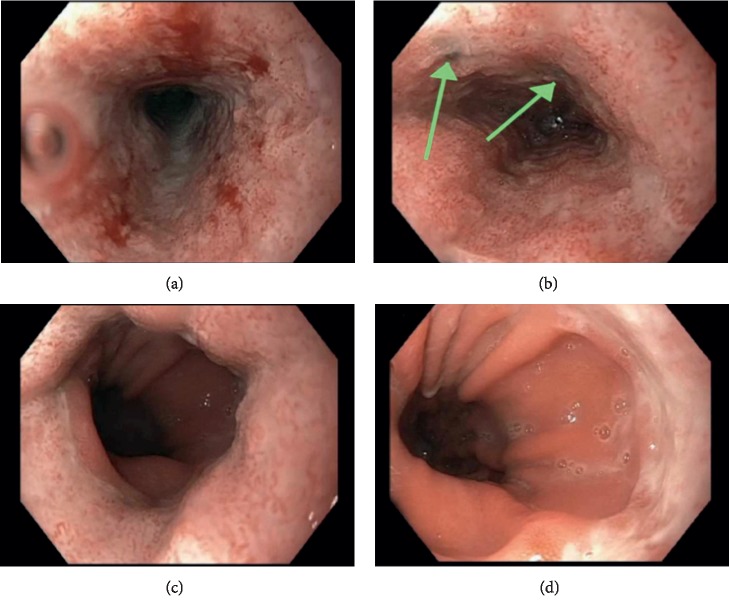
Upper gastrointestinal endoscopy. (a) Upper third of the esophagus. (b) Middle third of the esophagus shows areas of necrosis (green arrows). (c) Lower third of the esophagus. (d) Gastroesophageal junction.

Otorhinolaryngology was consulted and recommended a computed tomography (CT) of the neck with contrast, which did not reveal a retropharyngeal abscess. A modified barium swallow study showed no evidence of aspiration. Given the severity of her symptoms, gastroenterology was consulted. She underwent an esophagogastroduodenoscopy, which revealed Los Angeles Grade D esophagitis with two areas of esophageal necrosis and a large hiatal hernia (Figure 1). Biopsy of the stomach showed chronic gastritis and ulcerated mucosa with granulation tissue. CT chest, abdomen, and pelvis without contrast showed circumferential thickening of the mid-to-distal esophagus without evidence of esophageal rupture or leakage. The patient was started on pantoprazole, famotidine, and sucralfate with slow advancements in diet and was eventually able to tolerate oral intake. The patient was discharged home and instructed to follow-up outpatient with gastroenterology.

## 3. Discussion

AEN should be considered in critically ill diabetic patients with concerns for upper gastrointestinal bleeding, as gastrointestinal bleeding is the most common presentation of AEN [3–5]. AEN is a rare complication of DKA, as seen in other handful of cases [3–7], with the mechanism unknown. It has been suggested that poor nutritional status with hemodynamic instability, and hyperglycemia in DKA can lead to poor vascular flow and impaired mucosal barrier from corrosive injury of gastric content [3, 6]. Another theory is that osmotic diuresis in DKA leads to volume loss and hypoperfusion of the distal third of the esophagus given its watershed areas [6]. Our patient had evidence of esophageal necrosis in the middle third of the esophagus, which is less common. It is likely that the patient had lower esophageal involvement as well, but necrosis was not observed distally because it has either already dissipated or has yet to appear. Additionally, unlike the vast majority of other cases [2, 3, 5], she did not have any documented hypotensive episodes throughout her hospital course, which may suggest an alternate mechanism independent of blood pressure fluctuations. The combination of significant hypovolemia from DKA and osmotic diuresis as well as ongoing poorly controlled hyperglycemia may have impaired the intrinsic protective mechanism against gastric acid, making the esophagus more vulnerable. AEN cannot be diagnosed with imaging alone and is best diagnosed by direct visualization via esophagogastroduodenoscopy [2, 6]. Management involves treating the underlying insult, which includes fluid resuscitation and gastric acid suppression [3]. Given the risk of perforation due to impaired mucosal membrane, nasogastric tube insertion should be avoided [4]. Empiric antibiotics are not indicated unless there is a concomitant infection or a concern for esophageal perforation because antibiotics have also been reported to cause AEN [2–5]. Given that esophageal perforation and strictures are potential complications of AEN, imaging should be performed and surgical consultation should be made when perforation is suspected.

## 4. Conclusion

AEN is a potentially life-threatening complication of DKA. Although AEN's pathophysiological mechanism and relation of hyperglycemia are unclear, fluid resuscitation and gastric acid suppression are crucial to the management of AEN. Early consultation with gastroenterology should be made if AEN is suspected with diagnosis confirmed by direct visualization with esophagogastroduodenoscopy. AEN should be considered in patients who present with upper gastrointestinal bleeding and odynophagia after resolution of DKA, as AEN may be a more common complication of DKA than previously thought and may be underdiagnosed.
